# Slanted bilateral lateral rectus recession for convergence insufficiency-type intermittent exotropia: a retrospective study

**DOI:** 10.1186/s12886-020-01562-2

**Published:** 2020-07-14

**Authors:** Meiyu Ren, Qi Wang, Lihua Wang

**Affiliations:** Department of Ophthalmology, Shandong Provincial Hospital Affiliated to Shandong First Medical University, No.324, Jingwu Road, Jinan, 250021 Shandong Province China

**Keywords:** Intermittent exotropia, Convergence insufficiency, Surgery

## Abstract

**Background:**

The present study sought to investigate the efficiency and safety of slanted bilateral lateral rectus recession for the treatment of convergence insufficiency-type intermittent exotropia.

**Methods:**

This retrospective study included 34 patients who underwent slanted bilateral lateral rectus recession for convergence insufficiency-type intermittent exotropia in Shandong Provincial Hospital affiliated to Shandong First Medical University between September 2013 and October 2015 with a minimum follow-up of 6 months. A successful surgical alignment was defined as + 5 (positive for esotropia) to − 10 (negative for exotropia) prism diopters (PD) of orthotropia in the primary position while viewing distant or near targets and a near-distance deviation difference ≤ 8PD.

**Results:**

The mean age of the patients at surgery was 7.09 ± 3.80 years (range, 3 to 18 years). The mean distance deviations were − 26.09 ± 6.5 PD (range, − 15 to − 35 PD) and the mean near deviations, − 37.21 ± 6.3 PD (range, − 25 to − 45 PD) preoperatively. The mean recession amount of upper pole of the lateral rectus was 5.97 mm (range, 4.0 to 7.5 mm) and that of lower pole of the lateral rectus, 7.49 mm (range, 6.0 to 8.5 mm). At a mean follow-up of 15.0 months (range, 6 to 37 months), the surgical success rate was 70.6% (24/34), the under-correction rate was 17.6% (6/34), and the overcorrection rate was 11.8% (4/34). The mean near-distance deviation difference was significantly reduced from 11.12 ± 2.06 PD (range, 10 to 15 PD) preoperatively to 2.47 ± 3.04 PD (range, 0 to 10 PD) postoperatively (*P* < 0.001). Each millimeter of difference between the upper and lower poles of the lateral rectus recession was associated with an improvement of 5.65 PD in the near-distance deviation difference. At the final follow up, a near-distance deviation difference of ≤8PD was found in 32 (94.1%) patients. None of the patients developed A-V pattern, torsional diplopia, or restricted abduction of the eyes.

**Conclusions:**

Slanted bilateral lateral rectus recession may successfully reduce the distance and near exodeviations and the near-distance deviation difference, thus was proved to be an effective and safe procedure for the treatment of convergence insufficiency-type intermittent exotropia.

## Background

Convergence insufficiency-type intermittent exotropia (CI-IXT) is defined as exodeviation greater at near than at distance by at least 10 PD [1]. Patients of CI-IXT may have symptoms such as reading difficulty, blurred near vision, or diplopia sometimes. Non-surgical treatments such as orthoptic treatment, base-in prism reading glasses, vision therapy and psychotherapy could be used to alleviated symptoms in some patients [2]. Surgical management is required for patients that show little effect of non-surgical measures or for patients whose deviations are too large or poorly controlled [3]. The various surgical procedures for CI-IXT include unilateral or bilateral medial rectus (MR) resection(s), bilateral lateral rectus (LR) recessions, unilateral LR recession combined with MR resection [4–7], MR resection with adjustable sutures [3], slanted bilateral MR resections [8–10], improved unilateral LR recession combined with MR resection [11–13], and slanted bilateral lateral rectus recession (SBLR-rec) [1, 2, 14, 15]. The reported success rates range from 13.3 to 92%. Snir [1] proposed a new procedure of slanted unilateral or bilateral LR recession for both intermittent and constant types of CI-IXT in which the upper pole of the muscle was recessed according to the distance exodeviation and the lower pole was recessed according to the near exodeviation. It was reported that this procedure was superior in reducing both distance and near deviations and in reducing the near-distance (N-D) deviation difference. In another study performed by Kwon et al. [14], SBLR-rec was also proved to be safe and efficient in reducing the distance and near deviation and also the N-D deviation difference. However, Farid and Abdelbaset [15] compared SBLR-rec with two other surgical procedures and reported no significant differences among the three groups in the success rates of distance exodeviation, near exodeviation, and N-D deviation difference after 1 year. Moreover, they reported cases of asymptomatic vertical pattern strabismus after SBLR-rec procedure, while the other studies [1, 2, 14] didn’t report similar findings. In the present study, we retrospectively analyzed the surgical results of 34 patients who underwent SBLR-rec for CI-IXT with a minimum follow-up of 6 months.

## Methods

### Patients

In this retrospective study, the data of 34 patients with CI-IXT who underwent SBLR-rec between September 2013 and October 2015 were analyzed. Informed written consent for the surgical procedure was obtained from adults patients or parents or guardians of the children (<18 years) before surgery according to a protocol approved by the Medical Ethics Committee of Provincial Hospital affiliated to Shandong First Medical University for the protection of human subjects (No.2018–056). Inclusion criteria were as follows: the patients with CI-IXT (greater at near than at distance by at least 10 PD); best-corrected visual acuity in the worse eye 20/40 or better and with interocular difference of visual acuity no more than one line; deviation at distance between − 15 and − 35 PD by the prism and alternate cover test after proper optical correction; myopia or hyperopia <5.00D, astigmatism ≤2.00D (based on cycloplegic refraction) and anisometropia≤2.00D. Exclusion criteria were as follows: histories of strabismus surgery, myopic overcorrection, or convergence or fusion exercises; paralytic or restrictive strabismus; A or V pattern; oblique muscle overaction; vertical deviation greater than 5 PD; ocular disease other than strabismus; congenital anomalies or neurological disorders.

## Surgical procedure and data collection

Complete ophthalmologic and orthoptic evaluations were performed for each patient, including best-corrected visual acuity, cycloplegic refraction, anterior segment assessment, fundus examination, and motility evaluation. After proper optical correction, the deviations at distance (6 m) and near (33 cm) of each patient were measured by prism and alternate cover tests with fixation on accommodative targets. General or local anesthesia was performed according to the age or cooperation level of the patients. All surgeries were performed by the same surgeon. Following a conjunctival cul-de-sac incision, the muscle was disinserted, recessed and sutured directly to the globe. The upper pole of the muscle was recessed according to the distance exodeviation and the lower pole according to near exodeviation. Surgical doses for the LR recession were calculated primarily based on Park’s method [16].

Postoperatively, the follow-up visits were scheduled at 1 day, 6 weeks, 3 months and 6 months. As in our previous studies [13], alternate full-time patching, full cycloplegic hypermetropia prescription and base-out press-on Fresnel prism were adopted to deal with the patients overcorrected according to the angle of esodeviation and the duration of diplopia.

A successful surgical alignment was defined as an exodeviation (exophoria/ tropia) of − 10 PD or less and esodeviation (esophoria/tropia) of + 5 PD or less in primary gaze while viewing distant or near targets, and the N-D deviation difference ≤ 8PD. Under-correction was defined as postoperative exodeviations > − 10 PD or the N-D deviation difference > 8PD. and overcorrection were defined as postoperative esodeviation > + 5 PD.

### Statistical analysis

All analyses were performed with SPSS17.0 (StatLab, SPSS for Windows V.17.0). Paired t-test was used to analyze the preoperative and postoperative the N-D deviation differences. A *p* value of < 0.05 was considered statistically significant.

## Results

Thirty-four patients were enrolled in this study with 23 males and 11 females. The characteristics of the subjects were shown in Table 1. The mean age at surgery was 7.09 ± 3.80 years old (range, 3 ~ 18 years, median 6 years). The preoperative mean deviation at distance was − 26.09 ± 6.5 PD (range, − 15 ~ −35PD, median, − 25PD), while that at near was − 37.21 ± 6.3 PD (range, − 25 ~ − 45 PD, median, −40PD), with a N-D difference of 11.12 ± 2.06 PD (range, 10 ~ 15 PD, median, 10PD). A mean recession of the upper pole of LR muscle was 5.97 mm (range, 4.0 ~ 7.5 mm, median, 6 mm), and that of the lower pole of the muscle 7.49 mm (range, 6.0 ~ 8.5 mm, median, 7.5 mm). The mean follow-up period was 15 months (range, 6 ∼ 37 months, median,7).

**Table 1:** Characteristics of patients

	Mean ± SD (range)	Median
Age at surgery (years)	7.09 ± 3.80 (3 ~ 18)	6
male/female	23/11	
Equivalent spherical diopter (D, right)	−0.72 ± 1.29 (0 ~ − 4.00)	0
Equivalent spherical diopter (D, left)	−0.60 ± 1.20 (0 ~ − 4.00)	0
BCVA^#^ (LogMAR2^*^, right)	0.08 ± 0.13 (0.30 ~ 0)	0
BCVA (LogMAR, left)	0.09 ± 0.17 (0.30 ~ 0)	0
Deviation at distance (PD)	−26.09 ± 6.5 (−15 ~ −35)	−25
Deviation at near (PD)	−37.21 ± 6.30(− 25 ~ −45)	−40
N-D difference (PD)	11.12 ± 2.06 (10 ~ 15)	10
Follow-up period (month)	15.01 ± 11.19 (6–37)	7

#BCVA: best corrected visual acuity.

*LogMAR: logarithmic minimum angle of resolution

Table 2 shows the surgical outcomes of 34 patients with CI-IXT. At the last follow-up, the success rate was 70.6% (24/34), the under-correction rate 17.6% (6/34), and the over-correction rate 11.8% (4/34). The N-D deviation differences of 94.1% patients (32/34) were reduced to be ≤8 PD postoperatively. The mean N-D deviation difference was significantly reduced from 11.12 ± 2.06 PD preoperatively to 2.47 ± 3.04 PD postoperatively (*P* < 0.001) (Table 3). The mean deviation angle of 4 patients overcorrected was + 10.5 ± 3.0PD (range, + 6 ~ +12PD, median, 12PD) at distance and + 7.5 ± 5.7PD (range, 0 ~ + 14 PD, median, 8PD) at near, and the mean N-D deviation difference was 4.0 ± 1.6PD (range, 2 ~ 6PD, median, 4PD). One patient overcorrected with a deviation angle of +12PD at distance and + 14PD at near undertook a second surgery (unilateral medial rectus recession of 5 mm) 7 months after the first surgery and was successfully corrected. The mean deviation angle of 6 patients under corrected was − 10.7 ± 6.0PD (range, − 4 ~ −16PD, median, −12PD) at distance, and − 16.7 ± 3.0PD (range, − 14 ~ − 20 PD, median, −16PD) at near, and the mean N-D deviation difference was 6.0 ± 3.3PD (range, 2 ~ 10PD, median, 5PD). However, no second surgery was performed as each of them had a good control of the eye till last follow up.

**Table 2:** Surgical outcomes of patients with CI-IXT

	1 day	1 month	6 months	Last follow-up
Orthotropia (− 10, + 5) & N-D difference ≤ 8PD	10 (29.4%)	24 (70.6%)	24 (70.6%)	24 (70.6%)
overcorrection (> + 5PD)	23 (67.7%)	3 (8.8%)	5 (14.7%)	4 (11.8%)
undercorrection (> − 10PD) or N-D difference > 8PD	1 (2.9%)	7 (20.5%)	5 (14.7%)	6 (17.7%)

**Table 3:** The deviations and N-D differences before and after surgery (PD)

	pre-op	1d post-op	1 m post-op	6 m post-op	Last Follow-up
At distance	− 26.09 ± 6.5 (−15 ~ − 35)	8.38 ± 8.08	− 2.88 ± 6.50	−0.82 ± 6.17	−2.18 ± 6.85
Median at distance	−25	+ 10	0	0	0
At near	−37.21 ± 6.30 (−25 ~ − 45)	4.06 ± 9.39	−4.24 ± 7.08	−3.47 ± 6.10	−4.53 ± 7.65
Median at near	−40	0	−4	−2	−4
N-D Difference	11.12 ± 2.06 (10 ~ 15)	4.97 ± 4.48	3.24 ± 3.04	3.24 ± 3.41	2.47 ± 3.04
Median of N-D	10	5	4	4	1
P value (Pre & Po)*		< 0.001	< 0.001	< 0.001	< 0.001

^*^^P^-value: between the preoperation and the current angles at each time

Each millimeter of difference between the upper and lower poles of the LR recession was associated with an improvement of 5.65 PD (range, 0-10PD, median, 5.8PD) in the N-D deviation difference. Till the last follow up, A-V pattern, torsional diplopia, or restricted abduction of the eyes has been observed in none of the patients.

## Discussion

In recent years, various surgical procedures for CI-IXT include unilateral or bilateral MR resection(s) with or without adjustable sutures [3], slanted bilateral MR resections (the upper pole of the MR was resected according to the distance deviation and the lower pole was resected according to the near deviation) [8–10], improved unilateral LR recession combined with MR resection (the LR was recessed according to the distance deviation and the MR was resected according to the near deviation) [10–12], and SBLR-rec (the upper pole of the LR was recessed according to the distance deviation and the lower pole was recessed according to the near deviation) [1, 2]. Nemet first and then Biedner performed the slanted bilateral MR resections in 3 patients of CI-IXT respectively, and both reported fairly good outcomes [8, 9]. However, their studies had small samples, and 5 of the 6 patients had exodeviations at distance of <10PD. In another study of slanted bilateral MR resections conducted by Choi in 10 patients of CI-IXT, under corrections were found in all the patients both at distance and at near (with deviations of ≥10 PD) after a mean follow up of 38.9 months, only 50% of the patients (5/10) showed a N-D deviation difference of less than 10PD [10]. Kraft et al. performed improved unilateral LR recession combined with MR resection for CI-IXT (the LR was recessed according to the distance deviation and the MR was resected according to the near deviation), got a successful alignment both at distance and at near, and also decreased the N-D deviation difference [11]. Choi reported a success rate of 42.9% in a study of 14 children of CI-IXT with improved unilateral LR recession-MR resection after a follow-up of one year [12]. In our previous studies, we found that the improved unilateral LR recession-MR resection showed much better effect than unilateral or bilateral MR resections and may work better for CI-IXT patients [13]. Snir [1] adopted the procedure of slanted bilateral or unilateral LR recession for 12 patients (age range, 4–50 years) with CI-IXT and traditional lateral rectus recession for 6 patients with CI-IXT in the control group, and he reported a success rate of 92% in the study group, while in the control group all patients had residual exodeviations >8PD at near. In another study including 31 children with CI-IXT conducted by Chun and Kang [2], the success rate was reported to be 83.9%. And in Kwon and Lee’s study [14] in which 53 patients with CI-IXT underwent SBLR-rec with a follow-up of more than 12 months, the surgical success rate was 58.5%. Farid and Abdelbaset [15] compared the three surgical procedures for treatment of CI-IXT, which were, slanted bilateral LR recession(S-BLR), the improved unilateral MR resection-LR recession in which the resection were based on the near deviation and the recession on distance deviation, and augmented bilateral LR recession (A-BLR) in which the recession was based on the near deviation. However, after 1 year, the success rates among the three groups were reported to be statistically insignificant. But he also reported that in correcting distance and near exodeviation, the slanting recession procedure gained the highest success rate. In the present study, SBLR-rec was performed in 34 CI-IXT patients (age range, 3–18 years) and showed a success rate of 70.6%, which was lower than Snir’s [1] and Chun and Kang’s [2] studies, and higher than Farid and Abdelbaset’ study [15] and Kwon and Lee’s [14] study. Considering the factors such as sample size, age range, preoperative deviation angles, the definition of success, surgical techniques, follow up time and so on, the differences among the success rates were reasonable.

Although the success rates vary, the mean N-D deviation difference in these studies were mostly reduced significantly [1, 2, 14]. In our study, the mean N-D deviation difference was reduced from 11.12 ± 2.06PD preoperatively to 2.47 ± 3.04PD postoperatively. Each millimeter of difference between the upper and lower poles of the LR recession was associated with an improvement of 5.65 PD (range, 0-10PD, median, 5.8PD) in the N-D deviation difference. It proved that the slanted LR-rec procedure was effective in reducing the N-D deviation difference and thus an effective treatment for CI-IXT.

The present study indicated a prominent overcorrection rate (67.7%) 1 day postoperatively with a mean deviation angle of 8.38 ± 8.08PD at distance and 4.06 ± 9.39PD at near, which was similar to our previous study [13] and Kwon’s study [14] on surgical treatment of CI-IXT. After proper postoperative management such as alternate full-time patching and/or full correction of hypermetropia, the overcorrection rate decreased significantly in one month. After that, the mean deviations changed a small amount.

To explain the physiologic mechanism of the SBLR-rec for CI-IXT, Snir [1] considered that it was based on Scott’s investigation [17]. At the primary gaze, fibers length of the upper pole of LR muscle equals to that of the lower poles, 40.0 mm. While during down gaze and in near vision, the upper muscle fibers are lengthened by 1.5 mm and the lower muscle fibers are shortened by 2.9 mm. When the lower muscle fibers are weakened more than the upper fibers, the deviation at near would be reduced more than that at distance, resulting in a reduction of the N-D deviation difference.

In Farid and Abdelbaset’ study [15], at the final follow-up, 4 cases of 22 in the SBLR-rec group developed asymptomatic vertical pattern strabismus (V and A patterns) with no diplopia. However, in our present study and the other studies on slanted LR-rec procedures [1, 2, 14], none of the patients developed A-V pattern. Maybe more studies with larger sample and longer follow-up period are needed to prove the safety of the procedure.

The present study is limited by a few factors such as the retrospective nature, and the relatively short follow-up period. As over time, an exotropic drift would happen, which results in a further reduction of the surgical success rate. Even so, the study demonstrated that SBLR-rec procedure is effective in reducing the deviation angles of CI-IXT patients at distance and near, and also in reducing the N-D deviation difference.

## Conclusions

In summary, our data indicates that SBLR-rec for CI-IXT may successfully reduce the distance and near exodeviations and the N-D deviation difference, thus was proved to be an effective and safe procedure for the treatment of CI-IXT.

## Data Availability

The datasets of the current study are available from the corresponding author on reasonable request.

## Abbreviations

SBLR-rec: Slanted bilateral lateral rectus recession
CI-IXT: Convergence insufficiency-type intermittent exotropia
PD: Prism diopters
MR: Medial rectus
LR: Lateral rectus
N-D: Near-distance
BCVA: Best corrected visual acuity
LogMAR: Logarithmic minimum angle of resolution
S-BLR: Slanted bilateral LR recession
IRR: The improved unilateral medial rectus resection and lateral rectus recession
A-BLR: Bilateral augmented lateral rectus recession

## References

[1] Snir M, Axer-Siegel R, Shalev B, Sherf I, Yassur Y (1999). Slanted lateral rectus recession for exotropia with convergence weakness. Ophthalmology..

[2] Chun BY, Kang KM (2015). Early results of slanted recession of the lateral rectus muscle for intermittent exotropia with convergence insufficiency. J Ophthalmol.

[3] Choi DG, Rosenbaum AL (2001). Medial rectus resection(s) with adjustable suture for intermittent exotropia of the convergence insufficiency type. J AAPOS.

[4] Hermann JS (1981). Surgical therapy for convergence insufficiency. J Pediatr Ophthalmol Strabismus.

[5] Kushner B. Exotropic deviations: a functional classification and approach to treatment. Am Orthopt J. 1988; 38:81–93.

[6] von Noorden GK (1976). Resection of both medial rectus muscles in organic. Convergence insufficiency. Am J Ophthalmol.

[7] Burian HM, Spivey BE (1965). The surgical management of exodeviations. Am J Ophthalmol.

[8] Nemet P, Stolovitch C (1990). Biased resection of the medial recti: a new surgical approach to convergence insufficiency. Binocul Vis Strabismus Q.

[9] Biedner B (1997). Treatment of convergence insufficiency by single medial rectus muscle slanting resection. Ophthal Surg Lasers.

[10] Choi MY, Hwang JM. The long-term result of slanted medial rectus resection in exotropia of the convergence insufficiency type. Eye (Lond). 2006; 20:1279–83. 10.1038/sj.eye.6702095 16151478

[11] Kraft SP, Levin AV, Enzenauer RW (1995). Unilateral surgery for exotropia with convergence weakness. J Pediatr Ophthalmol Strabismus.

[12] Choi MY, Hyung SM, Hwang JM (2007). Unilateral recession-resection in children with exotropia of the convergence insufficiency type. Eye (Lond).

[13] Wang B, Wang L, Wang Q, Ren M (2014). Comparison of different surgery procedures for convergence insufficiency-type intermittent exotropia in children. Br J Ophthalmol.

[14] Kwon JM, Lee SJ (2019). Long-term results of slanted recession of bilateral lateral rectus muscle for intermittent Exotropia with convergence insufficiency. Korean J Ophthalmol.

[15] Farid MF, Abdelbaset EA (2018). Surgical outcomes of three different surgical techniques for treatment of convergence insufficiency intermittent exotropia. Eye..

[16] Parks MM. Ocular motility and strabismus. Hagerstown, MD: Harper & Row. 1975:99–111.

[17] Scott AB, Lenerstrand G, Bach-y-Rita P (1975). Strabismus muscle forces and innervation. Basic mechanism of ocular motility and their clinical implications: proceedings of the international symposium held in Wenner-Gren center, Stockholm.

